# Drug Resistance in Osteoarticular Tuberculosis: A Study From an Endemic Zone

**DOI:** 10.7759/cureus.44173

**Published:** 2023-08-26

**Authors:** Amartya Gain, Anil K Jain, Manpreet Bhalla, Aditya N Aggarwal, Ish K Dhammi, Vinod K Arora

**Affiliations:** 1 Orthopaedics, University College of Medical Sciences, New Delhi, IND; 2 Microbiology, National Institute of Tuberculosis and Respiratory Diseases, New Delhi, IND; 3 Pathology, University College Of Medical Sciences, New Delhi, IND

**Keywords:** anti-tubercular therapy, drug-resistant tuberculosis, mycobacterium tuberculosis, cartridge-based nucleic acid amplification testing, att, liquid dst, cbnaat, osteoarticular tuberculosis, drug resistance

## Abstract

Background: The present study was undertaken to determine the incidence of drug resistance against anti-tubercular drugs among patients from an endemic zone.

Methodology: Forty consecutive clinico-radiologically diagnosed patients of osteoarticular tuberculosis (29: spine, 11: extraspinal) were enrolled. Pus from needle aspiration was taken in 31 cases, tissue following spinal decompression in seven, synovial in one, and sinus edge biopsy in one. The pus/tissue was subjected to acid-fast bacilli (AFB) staining and liquid culture, sensitivity to 13 anti-tubercular drugs (Isoniazid (INH), rifampicin (RIF), kanamycin (KAN), amikacin (AMK,) capreomycin (CAP), ethionamide (ETH), levofloxacin (LEV), moxifloxacin (MOX), linezolid (LNZ), para-amino-salicylic acid (PAS), bedaquiline (BDQ), delamanid (DLM), and clofazimine (CFO)) were checked, and histopathological/cytopathological examination and molecular tests were performed.

Results: The mean age of patients was 29.07(9-65) years; 21 were female and 19 were male. The diagnostic accuracy for tuberculosis was 20% by AFB smear, 65% by liquid culture, 82.5% by histopathology, and 90% by cartridge-based nucleic acid amplification testing (CBNAAT). All culture-positive isolates were identified as *Mycobacterium tuberculosis* with no non-tubercular *Mycobacterium*. The drug resistance detected on CBNAAT was 11.1%, line probe assay (LPA) first line was 15.4%, LPA second line was 4%, and liquid drug susceptibility testing (DST) 11.5%. We detected 15.4% INH resistance, 11.1% RIF, 7.6% LEV, 3.8% MOX and PAS. No resistance was detected against second-line injectable drugs (SLID), ETH, LNZ, BDQ, DLM, and CFO.

Conclusions: No single laboratory modality can ascertain the diagnosis in all cases; hence, samples should be sent for all tests in tandem. In the presence of insufficient samples, tissue may be subjected to CBNAAT and histopathology to arrive at tissue diagnosis. In this subset, overall drug resistance incidence was 12.5% (5/40) with one patient each of isolated INH and RIF resistance, one of multidrug-resistance (MDR), and two of pre-extensively drug-resistant (pre-XDR). Primary drug resistance came out to be 11.1% (4/36) with one patient each of isolated INH and RIF resistance, one of MDR, and one Pre-XDR.

## Introduction

Drug-resistant tuberculosis (TB) continues to be a public health threat. The emergence of drug-resistant TB has threatened the goal of global TB elimination. Drug resistance in TB is categorized as single drug resistant-TB, multiple-drug-resistant (MDR)-TB, and extensively-drug-resistant (XDR)-TB [1]. Primary and acquired drug resistance incidence is well documented for pulmonary TB but not for osteoarticular TB (OATB) [2].

The rapid detection of drug-resistant TB improves treatment outcomes and prevents disease transmission [3]. Among OATB cases, suspected (presumptive) drug-resistant TB can be identified as patients showing failure of clinico-radiological improvement, deterioration of existing lesions, or the appearance of a fresh lesion/abscess while on anti-tubercular therapy (ATT) for a minimum of four to five months [4]. The drug sensitivity testing (DST) methods include culture-based (phenotypic) and nucleic acid-based (genotypic) methods such as GeneXpert® MTB/RIF (*Mycobacterium tuberculosis/rifampicin*) assay (Cepheid, Sunnyvale, California,, United States), cartridge-based nucleic acid amplification test (CBNAAT), and line probe assay (LPA) for first and second-line drugs.

The prognosis for spinal/extraspinal TB is improved by early diagnosis and intervention. Delay in diagnosis of drug-resistant spinal TB often results in the development of spinal deformity and/or neurological complications. There is a mean delay of six to eight months before the diagnosis of TB is ascertained, even in endemic countries, due to difficulty in procuring samples from deep-seated lesions as well as difficulty in demonstrating acid-fast bacilli (AFB) on smear and culture owing to the paucibacillary nature of the disease [5-7]. Genotypic DST can reduce this delay in establishing drug resistance and the institution of appropriate treatment [8,9].

The data highlighting the presence of drug resistance in a consecutive series of OATB cases is scarce. No data exist on the status of drug resistance against anti-tubercular drugs like bedaquiline (BDQ), delamanid (DLM), and clofazimine (CFO). We report the analysis of prospectively collected consecutive series of OATB cases investigated by molecular (CBNAAT and LPA), phenotypic (AFB smear and culture), and histological methods with the objective to determine drug resistance (incidence and the percentage) to 13 anti-tubercular drugs (isoniazid (INH), RIF, kanamycin (KAN), amikacin (AMK), capreomycin (CAP), ethionamide (ETH), levofloxacin (LEV), moxifloxacin (MOX), linezolid (LNZ), para-amino-salicylic acid (PAS), BDQ, DLM, and CFO, culture positivity rate, and micro-bacteriological profile (MTB complex/non-tubercular *Mycobacterium* (NTM) complex).

## Materials and methods

Forty consecutive patients of OATB (29 spinal and 11 extra-spinal cases) were enrolled between January 2021 and April 2022, with a mean age of 29 years (9-65 years) and a male:female ratio of 21:19. Patients diagnosed clinico-radiologically and on magnetic resonance imaging (MRI) observations were enrolled. Human immunodeficiency virus (HIV), hepatitis B surface antigen (HBsAg) or hepatitis C virus (HCV)-positive patients were excluded.

None of the patients in this study have been included in any previous studies and ethical clearance was obtained from the Institutional Ethics Committee - Human Research, University College of Medical Sciences, New Delhi, India (approval number: IECHR/2020/PG/47/31). Tissue samples were obtained by aspiration of cold abscesses (n=31), after surgical intervention (n=7), synovial biopsy of the knee (n-1), and sinus edge biopsy around the hip (n=1). Seven out of 29 spinal lesions were operated for debridement/decompression with/without instrumented stabilization. Pus, fluid, cartilage, synovial, inter-vertebral disc material, granulation tissue, caseous tissue, and bony tissue from the affected bone were sent for histopathology/cytopathology at our institute, and AFB smear, mycobacterial culture, CBNAAT, LPA, and liquid culture DST to 13 anti-tubercular drugs (INH, RIF, KAN, AMK, CAP, ETH, LEV, MOX, LNZ, PAS, BDQ, DLM, and CFO ) at the National Institute of Tuberculosis and Respiratory Diseases (NITRD), New Delhi, India. Standard operating procedures were followed during specimen collection, transport, and processing.

CBNAAT, if valid, was reported as MTB detected/not detected and sensitive/resistant to RIF. LPA was only done in culture-positive cases. First-line LPA was reported as MTB detected and sensitivity/resistance to RIF and INH was noted. LPA second line was reported as MTB detected and sensitivity/resistance to second-line injectable drugs (SLIDs) and fluoroquinolones (FQ) were recorded. In case of indeterminate result, the same test was repeated in the specimen available. The diagnosis of MTB was ascertained by various methods: molecular methods (CBNAAT, LPA), AFB smear, culture, and histology. The diagnostic accuracy of molecular methods and culture for MTB and the percentage of resistance to each 13 drugs were calculated. The diagnostic accuracy percentage of one modality alone was calculated and then with other modalities one by one. Once the diagnosis for MTB was ascertained, the standard treatment for different sites of OATB was followed with appropriate ATT (2HRZE + 10HRE) for sensitive disease and an appropriate regimen for proven drug resistance under the programmatic management of drug-resistant TB (PMDT) guidelines was instituted [1].

The data obtained from the laboratory reports was entered into a Microsoft Excel spreadsheet (2019; Microsoft Corporation, Redmond, Washington, United States) and analyzed.

## Results

The most common presenting symptom in our patients was pain, pain and swelling being the second most common presenting complaint. Constitutional symptoms (fever/night sweats/loss of appetite/weight loss) were present in 24/40 (60%) cases. Four patients out of 40 (10%) had a prior history of ATT intake before reporting to us for their existing condition.

Diagnostic accuracy

The diagnostic accuracy of AFB (Table 1) by smear examination using Ziehl-Neelsen (ZN) staining was 20% (8/40). The diagnostic accuracy on histopathology was 82.5% (33/40). The diagnostic accuracy of liquid culture using BACTEC Mycobacteria Growth Indicator Tube (MGIT) 960 System (Becton, Dickinson and Company, Franklin Lakes, New Jersey, United States) was 65% (26/40), with all positive cultures demonstrating MTB and no NTM isolated. The diagnostic accuracy for TB by CBNAAT was 90% (36/40). LPA test was done only in culture-positive cases (26); hence, comments cannot be made on the overall diagnostic accuracy of the test. However, the diagnostic accuracy for TB by LPA first line in culture-positive cases was 100% (26/26), and second-line LPA was 96.2% (25/26). The diagnostic accuracy of CBNAAT and LPA (in tandem) was 92.5% (37/40).

**Table 1:** The diagnostic accuracy of tests in Isolation and in tandem AFB: acid-fast bacilli; CBNAAT: cartridge-based nucleic acid amplification testing

Tests	Diagnostic accuracy
Smear for AFB	8/40 (20%)
Liquid Culture	26/40 (65%)
Cytopathology/Histopathology	33/40 (82.5)
CBNAAT	36/40 (90%)
Smear AFB + culture positive	27/40 (67.5%)
Smear AFB + culture + histology positive	37/40 (92.5%)
Smear AFB + culture + histology + Molecular tests positive	39/40 (97.5%)

Diagnostic Accuracy for TB for all Tests in Tandem

Diagnostic accuracy for AFB smear was 20% (8/40) cases, while for AFB smear and culture it was 67.5% (27/40. With addition of histology, the diagnostic accuracy increased to 92.7% (37/40). Upon addition of CBNAAT, the diagnostic accuracy rose to 97.5% (39/40) as the CBNAAT was diagnostic in remaining two cases. However, diagnosis could not be ascertained in one case when all tests were performed simultaneously possibly because the lesion was dry with no abscess; hence, little tissue could be procured on USG-guided aspiration.

Molecular tests

CBNAAT demonstrated RIF resistance in 4/36 (11.1%) cases. LPA first line showed drug resistance in 4/26 (15.4%) patients, out of which two patients were resistant to INH (7.7%) and two patients were resistant to both INH and RIF. LPA second line demonstrated resistance in 1/25 case against FQs. Genotypic DST (CBNAAT+LPA) showed: RIF resistance in 4/36 cases (11.1%), INH resistance in 4/26 cases (15.4%), FQ (LEV, MOX) resistance in 1/25 case (4%) (Table 2). SLID (KAN, AMK, CAP) resistance was not demonstrated in any case. Susceptibility to six drugs (PAS, ETH, LNZ, BDQ, DLM, CFO) could not be evaluated by genotypic DST since their molecular testing kit was not available. Liquid culture DST (phenotypic) showed: RIF resistance in 3/26 cases (11.5%), INH resistance in 3/26 cases (11.5%), MOX, LEV, and PAS resistance in 1/26 case (3.8%) separately (Table 3).

**Table 2:** Resistance pattern on molecular test drug sensitivity testing in participants INH: isoniazid; RIF: rifampicin; MOX: moxifloxacin; LEV: levofloxacin

Drug	No. of resistant cases	%
INH (n=26)	4	15.4
RIF (n=36)	4	11.1
MOX (n=25)	1	4
LEV (n=25)	1	4

**Table 3:** Resistance pattern on liquid culture growth finding in participants (n=26) INH: isoniazid; RIF: rifampicin; MOX: moxifloxacin; LEV: levofloxacin; PAS: para amino salicylic acid

Drug	No. of resistant cases	%
RIF	3/26	11.5
INH	3/26	11.5
MOX	1/26	3.8
LEV	1/26	3.8
PAS	1/26	3.8

KAN, AMK, CAP, LNZ, ETH, BDQ, DLM, and CFO resistance was not found in any cases. Overall drug resistance profile (Table 4) after combining genotypic and phenotypic DSTs showed INH 15.4% (4/26), RIF 11.1% (4/36), LEV 7.6% (2/26), MOX 3.8% (1/ 26), and PAS. No resistance was detected against SLID, LNZ, BDQ, DLM, and CFO. Overall drug resistance incidence was (12.5%) 5/40 cases (one patient each of INH and RIF mono-drug resistance, one MDR, and two Pre-XDR) with 4/5 having no prior history of ATT intake giving primary drug resistance 4/36 (11.1%).

**Table 4:** Overall drug resistance pattern combining both phenotypic and genotypic drug sensitivity testing INH: isoniazid; RIF: rifampicin; MOX: moxifloxacin; Lev: levofloxacin; PAS: para amino salicylic acid

Drug	No. of resistant cases	%
INH (n=26)	4	15.4
RIF (n=36)	4	11.1
LEV (n=26)	2	7.6
MOX (n=26)	1	3.8
PAS (n=26)	1	3.8

## Discussion

The prevalence of MDR, any drug resistance, and XDR in pulmonary TB is reported to be 3.5%, 24.9%, and 0.06% (among new) and 26.7%, 58.4%, and 1.3% (among previously treated), respectively [2]. However, no such data exists on OATB. Hence, in this study, we aimed to give the incidence of prevalent drug resistance including primary drug resistance in consecutive OATB patients. Since no single modality of laboratory investigation can ascertain the diagnosis of TB in all cases, hence pus/tissue obtained should be subjected to AFB smear/histology/culture and molecular tests simultaneously. We agree and reiterate the observation from two previous studies conducted by Abhimanyu et al. [10] and Yadav and Jain [11]

The observations analyzed on various tests in tandem are as follows:

(a) On conducting CBNAAT (36/40, 90%) and LPA in tandem, only one extra case could be diagnosed with a combined diagnostic accuracy of 92.5% (37/40); hence, the inclusion of LPA does not increase diagnostic accuracy significantly.

(b) On CBNAAT (90%) and liquid culture growth (65%) in tandem, the combined diagnostic accuracy became 92.5% (37/40). In one case, MTB was not detected on CBNAAT but was culture positive on MGIT.

(c) On molecular tests (CBNAAT and LPA) (92.5%) and cytopathology/histopathology examination (82.5%) in tandem, the combined diagnostic accuracy became 97.5% (39/40). In two cases, MTB was not detected on both CBNAAT and LPA but on tissue examination, these cases were consistent with TB.

Drug resistance

In our study, CBNAAT demonstrated RIF resistance in 11.1% (4/36) cases. Singh et al. reported 8.45% (6/71) RIF-resistant cases in his study while Abhimanyu et al. found RIF resistance to be around 4.62% (3/65) on CBNAAT [11,12].

First-line LPA demonstrated resistance to either INH or RIF in 15.4% (4/26). Only INH resistance in 7.7% (2/26) and both MDR (RIF and INH resistance) in 7.7% (2/26). Out of four INH resistance cases, two had low level and two had high level INH resistance. LPA second line demonstrated resistance in 1/25 case against FQs. In an unpublished study at our institute, Yadav and Jain reported two low-level and nine high-level INH resistance with either INH or RIF resistance in 25.5% (12/47), only RIF resistance in 2.1% (1/47), only INH resistance in 8.5% (4/47), and resistance to both RIF and INH (MDR TB) in 14.9% (7/47). Second-line LPA showed resistance to SLID in 1/36 and only FQ resistance in 5/36 cases [11].

In one case, RIF resistance was detected on CBNAAT and culture sensitivity; however, the LPA first line came out to be RIF sensitive. Neeraj et al. reported that such discordance between CBNAAT and LPA can be due to contamination, low bacterial loads, mixed organisms, and silent mutation [13].

DST interpretations

Genotypic DST (CBNAAT+LPA) to 13 anti-tubercular drugs showed: RIF resistance in 4/36 cases (11.1%), INH resistance in 4/26 cases (15.4%), and FQ (LEV, MOX) resistance in 1/25 case (4%). SLID (KAN, AMK, CAP) resistance was not demonstrated in any case. Susceptibility to six drugs (PAS, ETH, LNZ, BDQ, DLM, CFO) could not be evaluated by Genotypic DST since their molecular testing kit was not available.

Molecular kits only detect those resistances that have a known gene locus; therefore, resistances originating from mutations of other genes or gene regions as well as other unknown resistance mechanisms will not be detected [8]. These kits only screen the amino acid sequence and not the nucleic acid sequence so silent mutations will go undetected [14]. Therefore, solitary reliance on genotypic drug resistance detection would underestimate the disease burden and drug resistance rates [15].

Molecular tests cannot differentiate between members of the MTB complex. They are unable to differentiate between viable and nonviable bacteria DNA; hence, they cannot be used for monitoring the progression or success of treatment of patients with ATT [15].

Liquid culture DST (phenotypic) showed RIF resistance in 3/26 cases (11.5%), INH resistance in 3/26 cases (11.5%), MOX, LEV, and PAS resistance in 1/26 case (3.8%) separately. KAN, AMK, CAP, LNZ, ETH, BDQ, DLM, and CFO resistance were not demonstrated in any cases.

Culture-based or phenotypic DST testing can detect all known and unknown resistances irrespective of mutations and resistance mechanisms. It has low sensitivity since MTB is fastidious and difficult to grow [16-18]. These tests require high biosafety laboratory infrastructures and have a longer turnaround time of four to six weeks for detection of MTB with an added one to two weeks duration for DST, limiting its role in early diagnosis and interventions in drug-resistant TB [16-18].

Drug resistance profile after combining genotypic and phenotypic DSTs showed INH 15.4% (4/26), RIF 11.1% (4/36), LEV 7.6% (2/26), MOX 3.8% (1/26), and PAS. No resistance was detected against SLID, LNZ, BDQ, DLM, and CFO. LEV resistance was demonstrated in a total of two cases; one case was detected by second-line LPA and one additional case was detected by liquid culture. No additional case of MOX or SLID (KAN, CAP, AMK) resistance was detected on combining second Line LPA with liquid culture DST. On combining CBNAAT with first-line LPA, no new RIF-resistant case was detected by LPA. Detection of PAS resistance was exclusive to liquid culture DST and therefore no change was observed in combining genotypic DST with phenotypic DST.

Overall drug resistance incidence (patients who came drug resistant) was 12.5% (5/40 cases). Among these five cases, one patient was resistant to only INH, one was resistant to only RIF, one was MDR, and two were pre-XDR. Out of 40 patients, four patients had a history of ATT intake, and all of them took ATT for their existing condition. Out of the remaining 36 patients who had never taken prior ATT, four patients came out to be drug resistant (out of a total of five resistant patients); one patient each was INH, RIF MDR, MDR, and pre-XDR, giving a primary drug resistance (i.e drug resistance among patients who have never taken ATT) of 4/36 (11.1%).

Chen et al. reported 27.4% drug resistance with 15/113 (13.3%) as MDR and two XDR [19]. Hajiaheman et al. reported 15/40 (37.5%) cases as drug-resistant, of which six (15%) cases were single drug-resistant, nine cases (22.5%) were MDR, in which three cases (7.5%) were resistant to all first-line drugs as INH, RIF, streptomycin, and ETH [20]. However, both these studies included only those patients who were surgically intervened and not consecutive cases. There is no study that reported drug resistance incidence and profile of anti-tubercular drugs in consecutive cases of OATB like ours.

Limitations

The limitation of our study was the small number of enrolled cases. This was because the study was done during the coronavirus disease 2019 (COVID-19) pandemic when there was a low inflow of non-COVID-19 patients. However, since no data exists in the literature on the drug resistance parameters of consecutive cases of OATB patients, our study is worth reporting.

## Conclusions

Since no single modalities can ascertain diagnosis in all cases, hence samples should be sent for all the available tests: smear examination for AFB using ZN stain, TB culture, histopathology, CBNAAT, and LPA. However, an observation made in our study was that if an inadequate sample is obtained, tissue should be sent for CBNAAT and cytopathology/histopathology as combining them we got a diagnostic accuracy of 39/40 (97.5%). CBNAAT and liquid culture demonstrates drug resistance in most cases. Addition of LPA did not demonstrate extra RIF resistance cases. However, it did report an extra INH resistance case.

We found an overall drug resistance incidence of 12.5% (5/40) with primary drug resistance of 11.1% (4/36) in consecutive cases of OATB patients. Such high primary drug resistance in the existing population should give rise to a high degree of suspicion for drug resistance whenever we suspect a case of OATB and hence every effort must be made to procure pus/tissue from cold abscesses/pre/para-vertebral collection for DST (both molecular and phenotypic) so that ATT can be prescribed more effectively based on sensitivity pattern.
